# Supplementary material for: Magnetotransport on the nano scale
Source: Nat Commun. 2017 May 4;8:15283. doi: 10.1038/ncomms15283 (PMC5418608; doi:10.1038/ncomms15283)
Supplement: Supplementary Information — Supplementary Figures, Supplementary Table, Supplementary Notes and Supplementary References [file ncomms15283-s1.pdf]

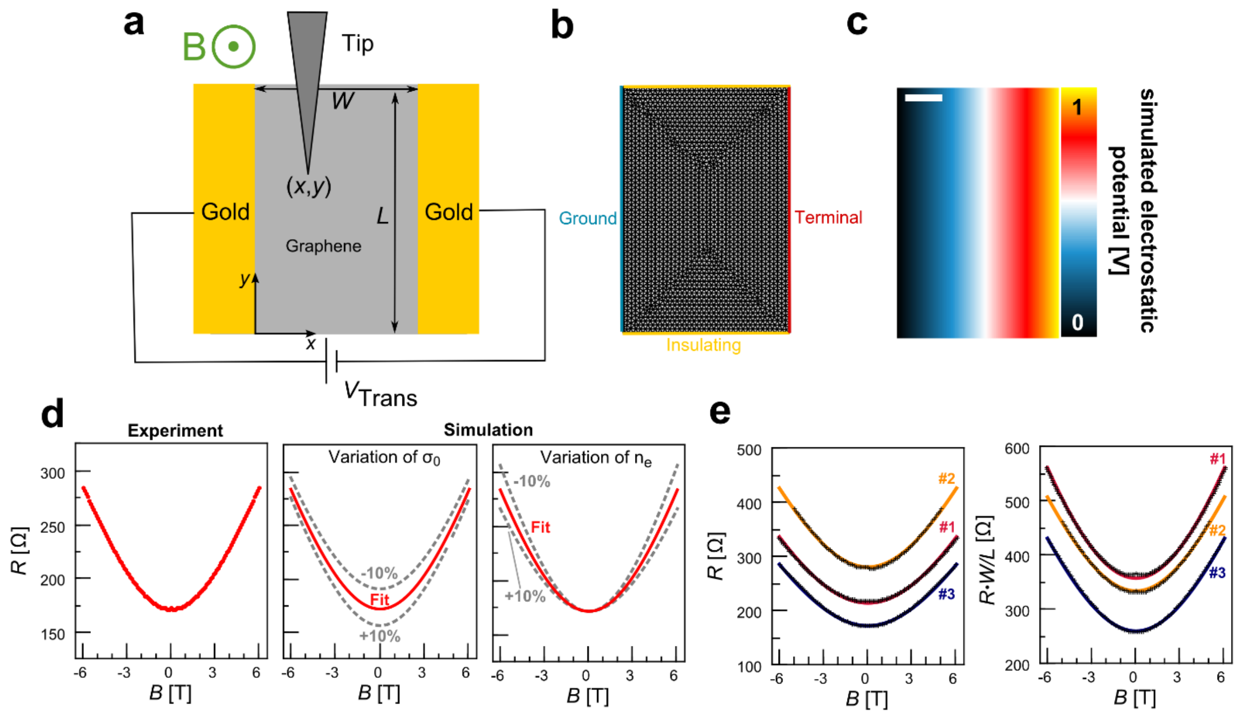

Supplementary Figure 1 │ Finite element simulations and fitting of MR-curves. a, The graphene sample of width *W* and length *L* is contacted in two-point geometry (*W*·*L*≈2-3 mm^2, see Supplementary Table 1). The coordinates (*x*,*y*) as used in the text refer to the position of the tip. **b,** Mesh grid and electrical boundary conditions. Black lines show the triangles used in the finite element simulations as initiated by COMSOL Multiphysics. The terminal is set to 1 V. **c,** Electrostatic potential obtained from the finite element simulation as shown in Fig. 1d in the manuscript (Scale bar 250 µm). **d,** Fitting of the experimental data to the finite element simulation. Left: Experimental MR for sample #3 in Supplementary Tab. 1, which is also shown in Fig. 1 in the manuscript. Middle: Fit of conductivity σ_0. Red line shows the best fit to the data with $\sigma_0 = 3.94$ mS; Grey lines show a 10% deviation from this value ($n_e = 1.25 \cdot 10^{13}\,\mathrm{cm}^{-2}$ for all curves). Right: Fit of the charge carrier concentration n_e. Red line shows the best fit to the data with $n_e = 1.25 \cdot 10^{13}\,\mathrm{cm}^{-2}$; Grey lines show a 10% deviation from this value ($\sigma_0 = 3.94$ mS for all curves). **e,** Total resistance R for all samples investigated in this study and the normalized resistance $R \cdot W/L$ as a function of magnetic field B. Black dots are experimental data and colored lines are fits done as sketched in d. Sample numbers correspond to those shown in Supplementary Tab. 1

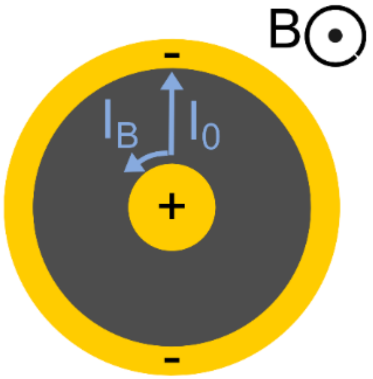

Supplementary Figure 2 │ Corbino disk contact geometry. Contacts are given by an inner and an outer circle while a transverse (perpendicular) magnetic field B is applied. For increasing B the current I_0 changes by the additional component I_B. Thus, the path an electron has to travel in the medium with resistivity ρ_0 increases leading to a positive MR.

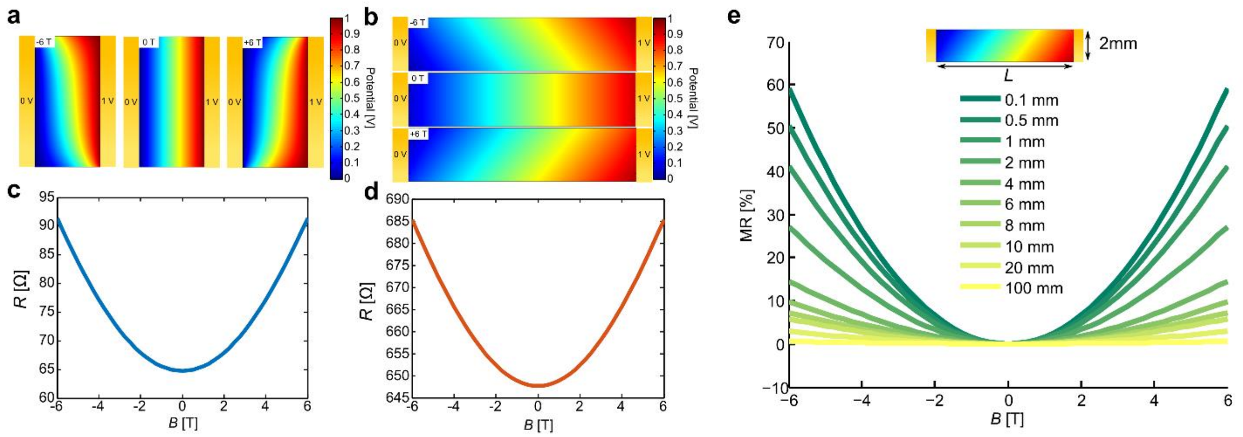

Supplementary Figure 3 | Potential distribution and Magnetoresistance for different contact geometries. a, Sample in short-channel geometry (MR geometry) with low ratio $L/W = 1\ \mathrm{mm}/2\ \mathrm{mm} = 0.5$. **b,** Long sample (Hall-geometry) with high ratio $L/W = 10\ \mathrm{mm}/2\ \mathrm{mm} = 5$. **c,** $R(B)$ for the sample geometry in a. **d,** $R(B)$ for the sample geometry in c. **e,** Magnetoresistance for different aspect ratios. Color-coded plots are simulated for different length L of the sample while the width $W = 2\ \mathrm{mm}$ is held constant. Thus, the aspect ratio L/W changes. Accordingly, for increasing aspect ratio the MR decreases. (Simulation parameters: $\sigma_0 = 7.84$ mS / $n_e = 1.23 \cdot 10^{13}\mathrm{cm}^{-2}$)

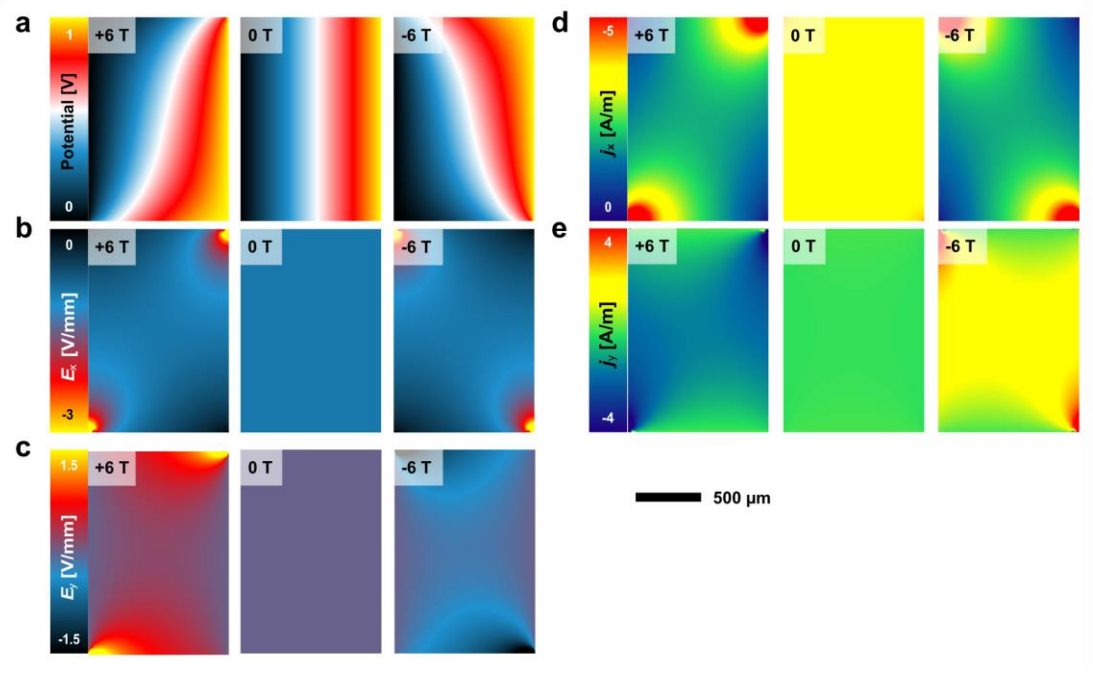

Supplementary Figure 4 │Macroscopic FEM simulations. a, Potential distribution as shown in Fig. 1d in the manuscript**. b,** Electric field component E_x. **c,** Electric field component E_y. **d,** Current density component j_x. **e,** Current density component j_y.

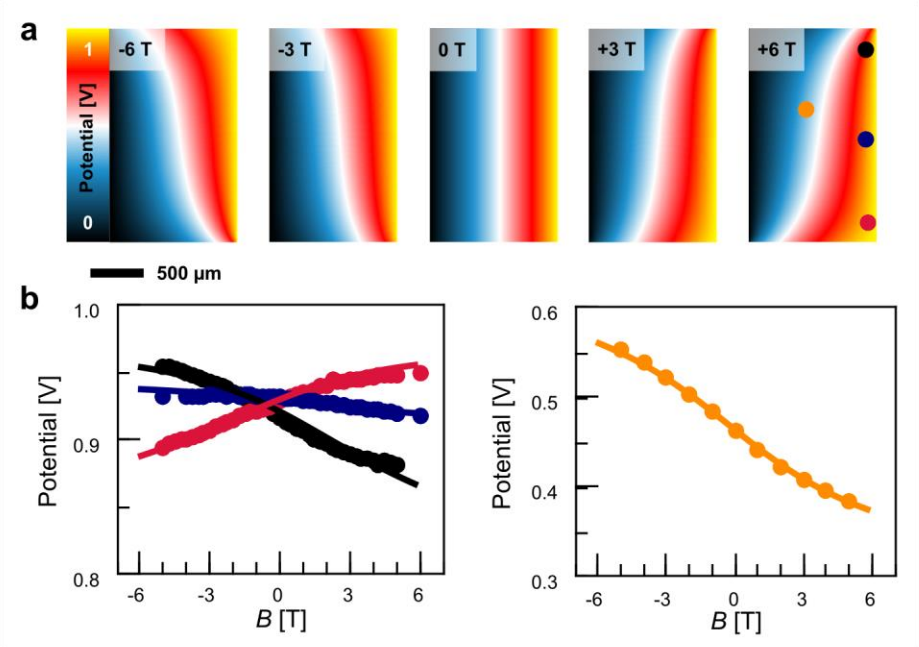

Supplementary Figure 5 │ Positioning via magnetic field-dependent potential shifts. a, Potential distribution as shown in Fig. 1d in the manuscript, but for sample #1 in Supplementary Table 1**. b,** Experimentally measured change in potential at the positions indicated in (a) with the respective curves from the simulation (solid lines). These positions of the tip can be roughly determined by an optical access within a range of $\approx 100\ \mu\mathrm{m}$. Within this experimentally determined range, the position-dependent, simulated V(B) curves have been further fitted to yield the best agreement with the experimental V(B). Thus, a very exact determination of the tip position is possible.

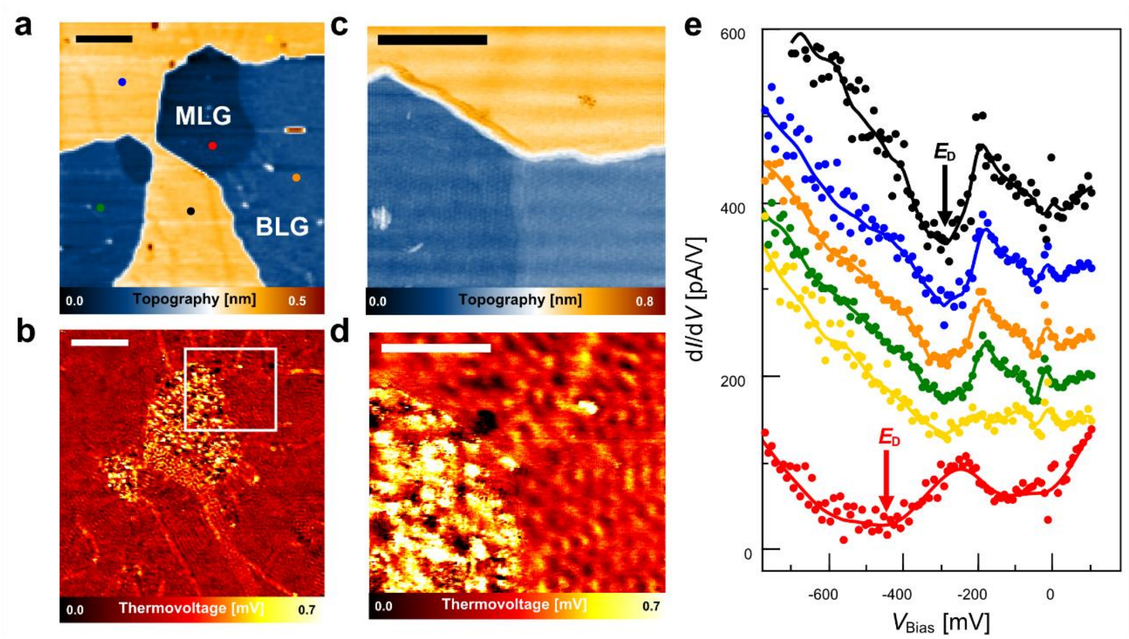

Supplementary Figure 6 | Scanning tunneling spectroscopy of monolayer and bilayer graphene. a, Topography of the sample region in Fig. 1c in the manuscript. **b,** Thermovoltage map. **c-d,** High resolution images of the upper right area in (a) and (b) [white square in b]. **e,** Scanning tunneling spectroscopy taken at the points indicated in (a). While the red line is taken on MLG, all other spectra are taken on BLG areas. The arrows indicate the position of the Dirac point E_{D}.

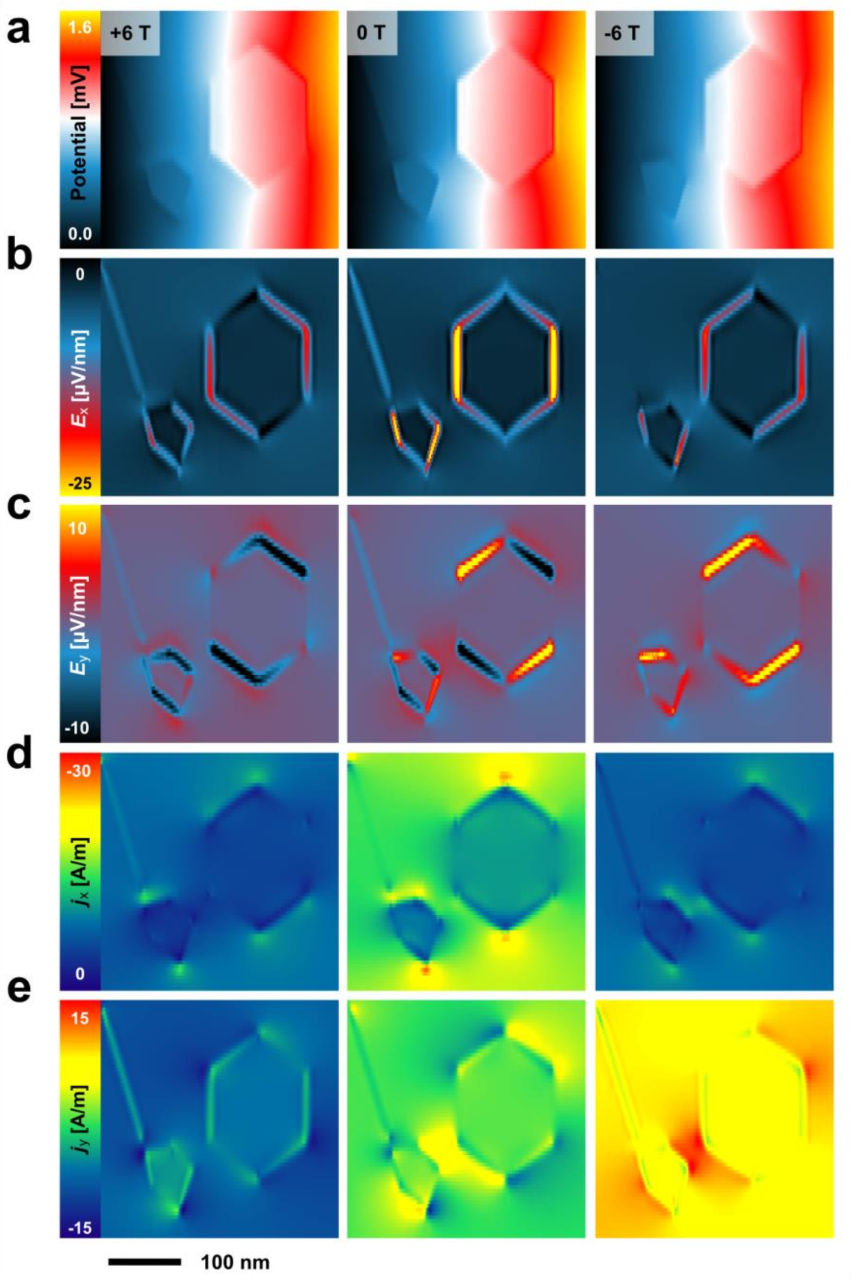

Supplementary Figure 7 │ FEM simulation for microscopic sample structure. Magnetic field dependent simulation for the sample topography in Fig. 1c in the manuscript with **a,** potential **b,** electric field E_x **c,** electric field E_y **d,** current density j_x and **e,** current density j_y.

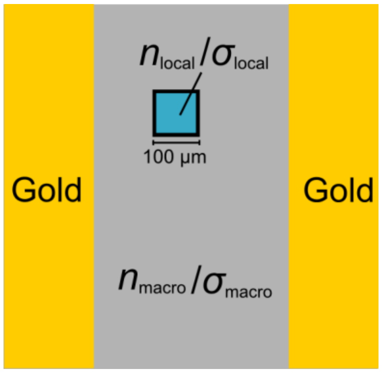

Supplementary Figure 8 | Geometry for FEM simulations to extract σ and n_e locally. For each sample geometry the conductivity tensor uses the macroscopic (average) values of σ and n_e as shown in Supplementary Table 1 (grey area). Locally both quantities are varied in a region of 100 µm x 100 µm (blue area). The position of that area fits that deduced from the potential analysis $V(B)$ for each dataset (see Supplementary Note 3). A small region (black area) of higher resistivity has been included to ensure continuity of current density in that sample region.

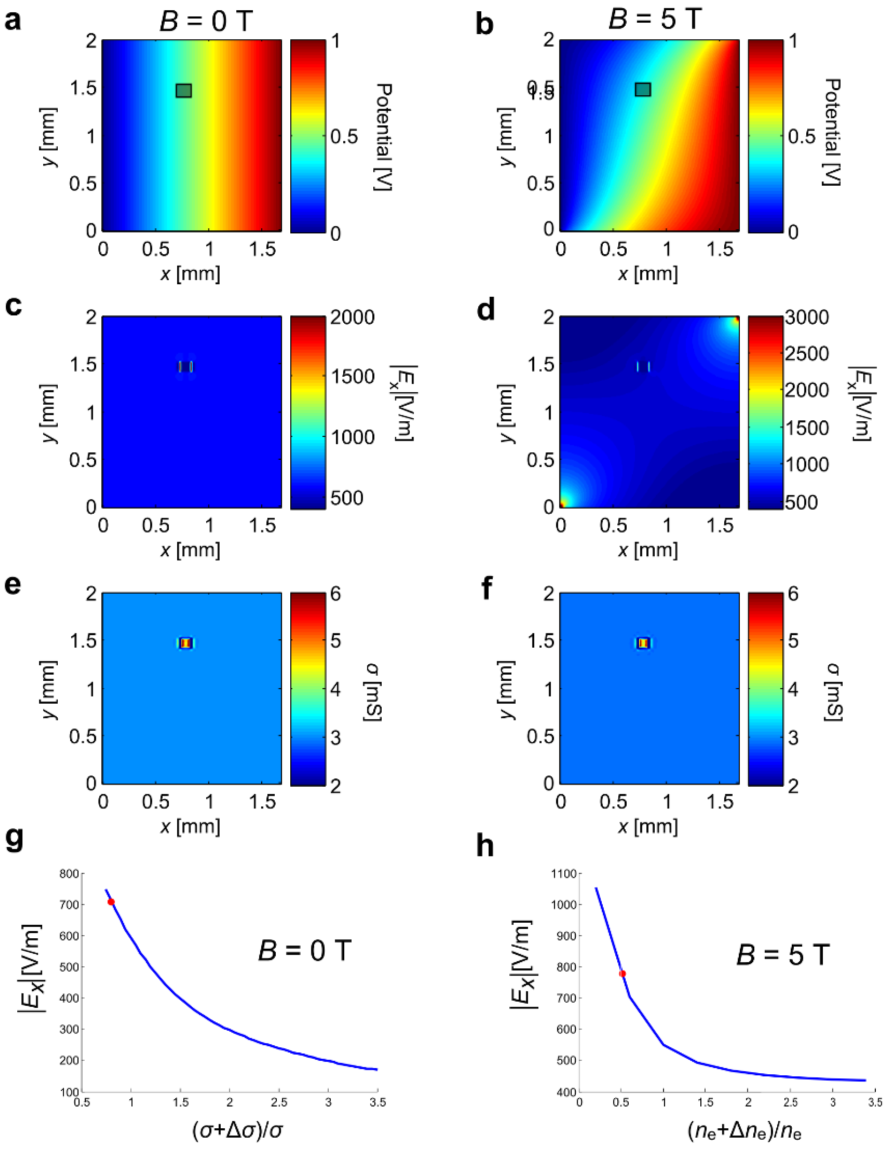

Supplementary Figure 9 │ Extracting σ and n_e from FEM simulations. Potential for **a,** 0 T and **b,** 5 T. In the indicated area the conductivity σ and the charge carrier concentration n_e have been increased by 50%. The corresponding electric field in x-direction and the calculated local conductivity are shown in **c-d,** and **e-f**, respectively. **g,** Fit of the electric field E_x as a function of local conductivity $(\sigma + \Delta\sigma)/\sigma$, 0 T. **h,** Fit of the electric field $\mathrm{E_x}$ as function of local charge carrier concentration $(n_e + \Delta n_e)/n_e$, , 5 T.

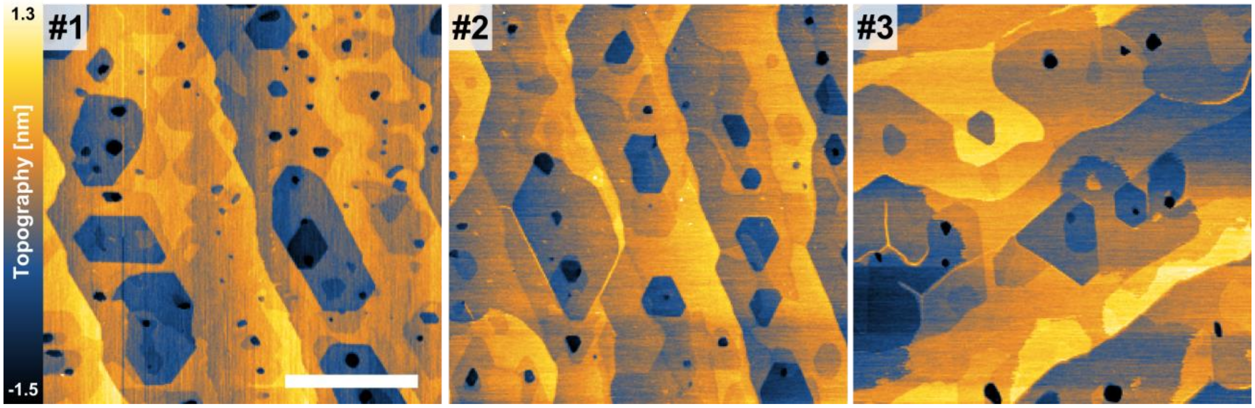

Supplementary Figure 10 | AFM topographies of all investigated samples. Sample No. is indicated in the upper left corner relating the images to the MR curves in Supplementary Figure 1e and to the values extracted in Supplementary Table 1 (Scale bar 1 µm). For all we find roughly an equal ratio of MLG/BLG-areas.

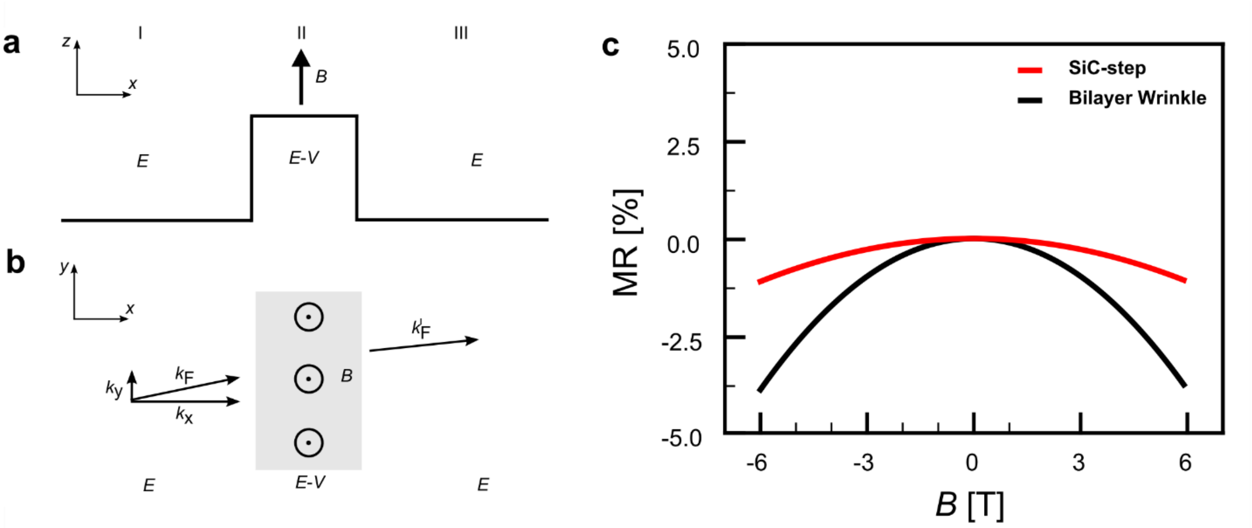

Supplementary Figure 11 | Transmission through a magnetic potential barrier. a, Potential barrier with an additional transverse magnetic field inside. **b,** Topview with components of the wave vector. **c,** Theoretical magnetoresistance for monolayer SiC-steps and bilayer wrinkles as a function of the magnetic field.

Sample No.	$W \times L$ [mm × mm]	$\langle\sigma\rangle$ [mS]	$\langle n_e\rangle$ [10^{13} cm^{-2}]	$\langle\mu\rangle$ [m^2/V · s]
**1**	2.1 × 1.2	2.65	1.07	0.155
**2**	2.0 × 1.7	3.02	1.09	0.173
**3**	1.6 × 1.1	3.94	1.25	0.197

Supplementary Table 1 | Sample dimensions, microscopically averaged conductivity $\langle\sigma\rangle$, averaged charge carrier concentration $\langle n_e\rangle$ and averaged mobility $\langle\mu\rangle$ for the macroscopic measurements for the samples used in this study.

Supplementary Note 1

Finite element method simulations of the macroscopic MR curves:

a) Derivation of the conductivity tensor

For a transverse magnetic field $\mathbf{B}$ perpendicular to the current flow $\mathbf{j} = -en_e\mathbf{v}_\mathrm{D}$ charge carriers get deflected by the additional Lorentz-force. Here, v_D is the drift velocity and n_e is the charge carrier concentration. This can be written as[1]

$$\frac{m}{\tau}\boldsymbol{v}_\mathbf{D} = -e[\boldsymbol{E} + \boldsymbol{v}_\mathbf{D} \times \boldsymbol{B}] \tag{1}$$

with m being the electron effective mass and τ the momentum relaxation time. Choosing $\boldsymbol{B} = (0,0,B)$ and solving for Ohm's law in the form $\boldsymbol{E} = \hat{\sigma}^{-1}\boldsymbol{j}$ in the limit of 2D transport yields[1]

$$\begin{pmatrix} E_x \\ E_y \end{pmatrix} = \begin{bmatrix} m/e\tau & -B \\ B & m/e\tau \end{bmatrix}\begin{pmatrix} v_x \\ v_y \end{pmatrix} = \sigma_0^{-1}\begin{bmatrix} 1 & \mu B \\ -\mu B & 1 \end{bmatrix}\begin{pmatrix} j_x \\ j_y \end{pmatrix} = \begin{bmatrix} \rho_0 & -R_H B \\ R_H B & \rho_0 \end{bmatrix}\begin{pmatrix} j_x \\ j_y \end{pmatrix} \tag{2}$$

Moreover, the Hall resistance $R_H = -1/en_e$ was introduced. The subscript for $\sigma_0 = e^2\tau\, n_e/m = 1/\rho_0$ indicates the zero-field conductivity/resistivity. Inverting the resistivity matrix yields

$$\hat{\sigma} = \frac{\sigma_0}{1 + (\mu B)^2}\begin{bmatrix} 1 & -\mu B \\ \mu B & 1 \end{bmatrix} \tag{3}$$

where $\mu = \sigma_0/e \cdot n_e$.

b) Resistor-Network simulation using COMSOL Multiphysics

The resistor network simulations have been conducted using finite element method (FEM) simulations with COMSOL Multiphysics (Version 5.2). Using the AC/DC module, in particular the electric currents toolbox, a simple rectangular sample has been simulated in a 2D space dimension (Supplementary Figure 1a). Width W and length L were taken from the actual samples geometries. Two sides (top, bottom) were set as electric insulation while the contacts were set as ground and a terminal as sketched in Supplementary Figure 1b. In addition to avoid numerical errors of device resistance, the mesh size has been set to *extra fine* ($< 40\ \mu\mathrm{m}$ edge length, Supplementary Fig. 1b). Thus, no changes with varying mesh size were found.

The magnetic field B_z in *z*-direction has been included by a manual input of the conductivity tensor in Supplementary Equation (3). Parametric sweeps for the B_z-field were done in the range between -6 T to +6 T. For further reading a detailed introduction on the simulation of Hall devices using COMSOL is given in Ref. 2. In addition to the resulting spatially resolved electrostatic potential (Supplementary Figure 1c) as well as electric field and current density components (See Supplementary Note 2), the total resistance $R(B)$ (magnetoresistance) of the device in Supplementary Figure 1a-c can be obtained from the simulation. To obtain the macroscopic (averaged over the whole sample) conductivity σ_0 and charge carrier concentration (CCC) n_e the simulated $R(B)$ curves were fitted to the experimental ones. Supplementary Figure 1d shows the experimental MR curve for sample #3 also shown in Fig. 1 in the manuscript along with simulations for different values of σ_0 and n_e. For $B = 0$ only σ_0 influences the total

resistance and can thus be deduced (Supplementary Figure 1d, middle, this is also for rectangular samples simply given as $\sigma_0 = R(0)^{-1} \cdot L/W)$). In addition, for $B > 0$ the CCC n_e changes the MR and can thus additionally be determined (Supplementary Figure 1d, right). In Supplementary Figure 1e, left fits to all samples of this study are shown. While the difference in offset and slope of the curves stems on the one hand from variations in width W and length L of the samples [See also discussion in Supplementary Note 1(c)] it is on the other hand also a result of different defect densities of steps and interfaces (influencing σ_0, see also Supplementary Figure 10) and different ratio between monolayer and bilayer graphene (influencing σ_0 and n_e). The fitted values for all samples are shown in Supplementary Table 1. For better comparison of the samples Supplementary Figure 1e, right shows the normalized resistance $R \cdot W/L$ which is the sheet resistance $\rho_{\text{sheet}} = 1/\sigma_0$ for $B = 0\ \text{T}$.

c) Geometry-dependence of the magnitude of the magnetoresistance

As discussed in the manuscript the geometry of a device has a crucial influence on the absolute value of the total resistance $R(B)$. A geometry-related increase of MR is nicely visualized in the geometry of a Corbino disk[3,4] (Supplementary Figure 2) and it should not be confused with a B-field dependency of the diagonal element of the conductivity tensor. For no magnetic field, the electrons can directly flow from the inner to the outer contact, while for finite field they get deflected by an angular component. Due to the radial symmetry, they still move in the outside radial direction, but cannot travel the shortest current path. Therefore, more scattering events occur and $R(B)$ increases. Since for this geometry the electric field components do not change under the influence of a magnetic field, the change in MR[3]

$$R(B) = R_0 \cdot [1 + (\mu B)^2] \tag{4}$$

can directly be seen from the denominator of the prefactor in Supplementary Equation (3). Now, this geometric MR can be more or less pronounced depending on the sample geometry. For example, in a Hall geometry (e.g. Supplementary Figure 3b) the Hall field completely compensates the Lorentz force leading to $R(B) \approx R_0$.

Supplementary Figure 3 shows simulations for different sample geometries in case of no magnetic field as well as for $\pm 6\ \text{T}$. In the spatially resolved potential images in Supplementary Figure 3a the contacts induce boundary conditions, since they are on a fixed potential and thus the equipotential lines in their vicinity are heavily bent for applied magnetic field B: Electrons are deflected in the sample due to the Lorentz force and pile up on one side causing a voltage V_H in y-direction. In contrast to the Corbino disk the existence of a Hall field can partially compensate for the magnetic forces and thus the geometric MR. However, unless the aspect ratio of a Hall geometry is reached (Supplementary Figure 3b) the sample still shows a geometric MR as in Supplementary Equation (4) induced by the contact regions. Here, the deflection is not fully compensated as in the case of the Corbino disk making the contact regions responsible for the observed MR (An extended discussion on this issue is given in Ref. 5). Therefore, short-channel samples with L<W (Supplementary Figure 3a) are dominated by the contact regions and show a strong MR (Supplementary Figure 3c, relative increase ≈50%). In contrast, for long thin geometries with L>W shown in Supplementary Figure 3b (Hall-geometry) this

contribution vanishes, since a constant electric field gradient E_y in y-direction has been established here and electrons are no further deflected because V_{H} compensates the Lorentz force. The respective MR-curve is shown in Supplementary Figure 3d with almost no dependence on magnetic field (obey different y-scale, relative increase ≈5%). Supplementary Figure 3e quantifies this observation demonstrating that the magnitude of the observed MR is especially present for short samples and is vanishing in the limit of perfect Hall geometries.

In the framework of this study, the geometry was on purpose chosen to be short-channeled (Supplementary Figure 3a) instead of using a Hall-geometry (Supplementary Figure 3b). In this way, the magnetic-field independent behavior of the defect resistances was easier to distinguish from the change in electric fields on the sheets.

Supplementary Note 2

Inhomogeneity of local current densities and electric fields: In a rectangular shaped sample as shown in Supplementary Figure 1a the current density components j_x, j_y as well as the electric field components E_x, E_y cannot be assumed to be homogeneous under the influence of a magnetic field. This is demonstrated in Supplementary Figure 4 for the geometry of sample #3 that is also shown in Fig. 1-3 in the manuscript. Especially at the corners of the sample the electric fields and current densities increase/decrease drastically. As a consequence the values in the center are also smaller than could be expected from e.g. simply calculating $j_x = V_{\mathrm{Trans}}/W \cdot R(B)$ for the current density in x-direction, where *R(B)* is the total resistance of the sample as a function of magnetic field *B*.

Supplementary Note 3

Determination of the tip position from potential measurements: The current density j is not uniform for an applied magnetic field (Supplementary Figure 5). However, since this value is needed to extract e.g. the defect resistance $\rho_{\mathrm{Defect}} = \Delta V / j_x$ we developed a method to determine the tip's position. Then the current density at this position is estimated by the resistor network simulations. In the experiment the approximate position of the tip can be obtained via an optical access. However, we determine the position of the tip on the sample more precisely by the change in potential *V(B)* with magnetic field. While the potential varies only along the x-direction for 0 T, it also changes in y-direction for a nonzero magnetic field. Therefore, the x-position of the tip on the sample can be determined with $B = 0\ \mathrm{T}$ while its change with magnetic field determines the y-position. This is demonstrated for different positions of the tip on the sample (Supplementary Figure 5a) in Supplementary Figure 5b with excellent agreement between experiment and simulation. Thus, by comparing the potential as a function of magnetic field with the simulations the position of the tip can be extracted.

Supplementary Note 4

Charge carrier concentration on ML and BL graphene: From the Hall field shown in Fig. 2b and evaluated in Fig. 2d in the manuscript we can extract the charge carrier concentration. The electric field component E_y in an arbitrarily rectangular sample is given by[6]

$$E_y(x,y,B) = [4 \cdot Q(x,y,W,L)/\pi] \cdot E_{Hall} \quad \text{where} \quad E_{Hall} = j_x B/qn_e \qquad (5)$$

Here, $Q(x,y,W,L)$ is a quality factor that only depends on the samples width W and length L as well as the coordinates *x/y* of the probe measurement. The latter can be obtained by the average change in potential as a function of B, which is unique for every point of the sample (See Supplementary Figure 5 and Fig. 2c in the manuscript). $E_y(x,y,B)$ for the position in Fig. 1c,e in the manuscript is shown in Fig. 2e in the manuscript. For this position the charge carrier concentration can be calculated by

$$n_e = [4 \cdot Q(x,y,W,L)/\pi] \cdot j_x(B) \cdot B / E_y(x,y,B)/e \approx (1.32 \pm 0.12) \cdot 10^{13}\ \mathrm{cm}^{-2} \qquad (6)$$

where we took $j_x(B)$ again from the resistor network simulations. The error stems from the uncertainties of the fit to $E_y(x,y,B)$ in Fig. 2e in the manuscript.

Besides the Hall measurements, the charge carrier concentration can additionally be determined by Scanning Tunneling Spectroscopy (STS) as shown in Supplementary Figure 6. By using thermovoltage imaging[8] in Supplementary Figure 6b,d we can distinguish between MLG and BLG. While BLG areas show standing wave patterns, MLG areas show a disordered electronic signature due to inhomogeneities in the buffer layer. In Supplementary Figure 6e we show STS measurements from different positions indicated in Supplementary Figure 6a. For the BLG areas we find the position of the Dirac point at E_D=(-300±20) mV, while for MLG it is located at E_D=(-450±30) mV. This is in excellent agreement with ARPES data on these systems that extracted charge carrier concentration of $n_{MLG}=1.0\cdot10^{13}$cm^{-2} and $n_{BLG}=1.3\cdot10^{13}$cm^{-2} for these energetic positions of the Dirac point[9].

Since the surface area in Supplementary Figure 6a is mostly covered by BLG, this agrees well with the value obtained by the local Hall measurement.

Supplementary Note 5

Simulation of transport on the nano scale including local defects: For the simulation of the electric fields of the microscopic sample structure in Fig. 2a and Fig. 2b in the manuscript we used the same resistor network method as described in Supplementary Note 1. The defects have been modeled by an isotropic conductivity tensor of $\sigma_{\mathrm{ML/BL}} = 0.05 \cdot \sigma_0$ and $\sigma_{\mathrm{Wrinkle}} = 0.3 \cdot \sigma_0$ and a transition width of 5 nm. These values yield the same zero-field defect resistances as shown in Tab. 1 in the manuscript. To minimize the number of parameters we set $\sigma_{\mathrm{ML}} = \sigma_{\mathrm{BL}} \approx \sigma_0$, since both show similar values (See Tab. 1 in the manuscript). For them the conductivity tensor in Supplementary Equation (3) was used.

The complete simulation of the microscopic sample structure can be found in Supplementary Figure 7. Here, we additionally included the current densities j_x/ j_y and the potential besides the electric field components E_x/ E_y shown in the manuscript.

Both experiment and simulations demonstrate how the influence of the magnetic field changes the current flow and enhances or depletes the electric field on the different facets of the large MLG hexagon. Consequently, the local voltage drop is in first approximation

well-described by a semi-classical model. The resistance of our defects does not show an explicit dependence on magnetic field or the angle of incidence of the electrons as could be expected from quantum mechanical effects in graphene, e.g. Klein tunneling[7].

Supplementary Note 6

Evaluation of magnetotransport data: Here, we describe how to locally extract the sheet conductivity σ and charge carrier concentration n_e (part I) as well as the absolute defect resistance ρ_{Defect} and its change with magnetic field (part II) as shown in Fig. 4e-f in the manuscript. The measured local electric fields $E_x(B)$ (sheets) and voltage drops $\Delta V(B)$ (defects) are needed as experimental input. These are evaluated as averaged sections as shown in Fig. 4d in the manuscript. Prior to that, the raw data has been evaluated as described in Ref. 8 to eliminate thermovoltage contributions.

a) Conductivity and charge carrier concentration for MLG and BLG

To extract the information about the MLG and BLG sheets we compared the experimentally observed electric fields $E_x(B)$ to finite element simulations.

For each dataset taken at a certain position of the sample simulations have been conducted changing both σ and n_e locally. This is necessary, since the change in field $E_x(B)$ is varying across the sample in the presence of a magnetic field (See Supplementary Figure 4c). The position of each dataset has been deduced from the change in the local potential $V(B)$ as demonstrated in Supplementary Note 3.

Supplementary Figure 8 shows the simulated geometry. For the majority of the sample the macroscopic average values for conductivity $\langle\sigma\rangle$ and charge carrier concentration $\langle n_e\rangle$ are used (See Tab. 1 in the manuscript and Supplementary Table 1). At the position where the experimental data was taken, both conductivity σ and charge carrier concentration n_e are varied in the simulation. A boundary region of lower conductivity was used to keep the current density constant across the sample. An area of 100 µm x 100 µm was chosen which is reasonably large to ensure no boundary effects from the transition region and is still numerically feasible as well. This simulated geometry mimics the experimental situation that the voltage drop on the MLG and BLG sheets is different from the effective, macroscopic voltage drop which emerges from a mixture of defect scattering and sheet resistance.

Subsequently, the electric field $E_{x,\text{sim}}(B)$ at this position has been simulated for different local conductivity σ and charge carrier concentration n_e in the area. Both quantities affect the conductivity tensor in Supplementary Equation (3) by changing $\frac{\sigma}{[1+(\mu B)^2]}$ as well as the off-diagonal terms. $E_{x,\text{sim}}(B)$ is next compared to the experimental value $E_{x,\text{exp}}(B)$ yielding the best fit of σ and n_e.

The evaluation is demonstrated in Supplementary Figure 9. Supplementary Figure 9a,b show the macroscopic potential across sample #2 for 0 T and 5 T, respectively. Additionally, the electric field in x-direction $E_x(B)$ is shown in Supplementary Figure 9c,d. Here, σ and n_e have been altered at the position of the dataset shown in Fig. 4a-d in the manuscript. Both n_e and σ have been increased by 50% (arbitrary choice).

The consistency of the simulations can be checked as shown in Supplementary Figure 9e,f by additionally calculating the sheet conductivity by

$$1/\sigma = E_{||}(B)/j(B) \quad (7)$$

where $j = \sqrt{j_x^2 + j_y^2}$ is the total current density and $E_{||}$ is the electric field in direction of j. Thus, the sheet conductivity σ can already be obtained from zero field measurements via the measured electric field $E_x(0\ \mathrm{T})$ and the current density $j_x(0\ \mathrm{T}) = j(0\ \mathrm{T})$. Subsequently we use the magnetic field measurements to determine the charge carrier concentration n_e.

Supplementary Figure 9g,h show the change in $E_x(\sigma)$ for 0 T and $E_x(n_e)$ for 5 T, respectively. The dots represent the experimentally measured values for the large MLG area in Fig. 4a in the manuscript and allow to deduce σ and n_e.

b) Defect resistance as a function of B

The defect resistance of all defects is calculated by

$$\rho_{\mathrm{Defect}} = \Delta V(B)/j_x(B) \quad (8)$$

Here, the voltage drop $\Delta V(B)$ is obtained experimentally and the local current density $j_x(B)$ is taken from the simulations.

We are aware that in the analysis the inhomogeneities in current density will also be present on a local scale as suggested by Fig. 3 in the manuscript and Supplementary Figure 7. This has to be taken into account for a quantitative analysis of local sheet resistances and defect line resistances.

Nevertheless, since the local current density yields on a larger scale (500 nm) the correct macroscopic value (Fig. 2 in the manuscript), we approach the problem by averaging out the current density inhomogeneities by a sufficiently large number of data sets from different positions of the sample. In total we analyzed 32 datasets for MLG sheets, 47 for BLG sheets, 34 for ML/BL-interfaces, 29 for wrinkles and 3 for SiC-steps from 12 positions on 3 samples. Note that this includes datasets from the same sheet/defect, but different magnetic field B.

Supplementary Note 7

Discussion on local sheet conductivity and defect resistance: The results of the analysis discussed in the last section and already plotted in Fig. 4e-f in the manuscript are shown again in Tab. 1 and 2 for the MLG and BLG sheets as well as for the defects.

For the MLG and BLG sheets the conductivity σ is higher than macroscopically observed, which obviously stems from the fact that the macroscopic conductivity still contains the influence of local defects. The higher conductivity σ of the bilayer compared to the monolayer can be explained with the higher charge carrier concentration n_e as well as smaller influence of the underlying buffer layer inducing additional scattering. It is however not twice the monolayer value, since only one of the two bilayer bands is populated at the given doping level.[9] The large standard deviation $\Delta\sigma$ can be caused by changes in current density $j_x(B)$ that has to be taken from the FEM simulation and cannot be measured locally as discussed above. This would lead to incorrect values calculated by Supplementary Equation (7), since inhomogeneities, e.g. the presence of extended defects, can cause a

locally higher or lower current density[10,11]. While the statistics assures that these are averaged out, the variations also arise from the properties of the sample system as already discussed in the main text: local variations in mobility μ and charge carrier concentration n_e lead to a large standard deviation $\Delta\sigma$ for the conductivity σ. This is supported by the additional large standard deviation Δn_e found for n_e. Additionally, we found previously large variations in conductivity for evaluations by resistor network simulations taking account for the variations in the local current density[10]. Mobility variations can be caused by inhomogeneities in the graphene buffer layer as shown in Supplementary Figure 6b,d. This also affects n_e, since the buffer layer is influencing the doping level[12]. Additionally, stacking faults in bilayer graphene[13] can induce additional strain and differently stacked bilayer both influencing the mobility/conductivity.
Despite the large $\Delta\sigma$ and Δn_e caused by locally varying characteristics of the sample, the errors of the average value of σ and n_e remain small due to the large number of data points taken.
The role of the defects in the macroscopic measurements can be estimated by large-scale AFM measurements of all investigated samples as shown in Supplementary Figure 10. The defect density of steps and interfaces is decreasing with sample number. In contrast, the macroscopic average conductivity is increasing as can be seen from Supplementary Table 1.

Moreover, for short length scales the definition of a local conductivity is only well-defined, if the electron scattering length is small compared to the distance between steps and interfaces and for inhomogeneities in the local charge carrier concentration caused by the buffer layer. In other words, a classical conductivity is only well-defined in a diffusive regime when transport is not ballistic. Other transport studies investigated this transition in greater detail. Jobst et al. found a transport time of $\tau = \left(\hbar\sqrt{\pi n_e}/e v_F\right) \cdot \mu = 0.045\ \mathrm{ps}$ (derived from their Drude resistivity) in case of quasi-freestanding monolayer graphene on SiC[14]. Using the values from Table 1 and 2 in the manuscript ($n_e \approx 1 \cdot 10^{13}\mathrm{cm}^{-2}$, $\mu \approx 0.2\ \mathrm{m}^2/\mathrm{V}\cdot\mathrm{s}$) and $v_F \approx 1 \cdot 10^6\ \mathrm{m}/s$ yields $\tau \approx 0.07\ \mathrm{ps}$ in good agreement with the above stated value. This can also be converted into units of length by $L = \sqrt{D \cdot \tau}$ with the diffusion constant $D = (n/e \cdot N_s) \cdot \mu$ and N_s being the density of states[1]. For monolayer graphene with $N_s(E) \approx (2/\pi[\hbar v_F]^2) \cdot |E|$ this yields $L \approx 50\ \mathrm{nm}$. Within weak-localization analysis even lower values have been found for the intravalley scattering time ranging between $10 - 20\ \mathrm{nm}$[15,16]. This is smaller than the typical distance between steps and interfaces ($> 100\ \mathrm{nm}$). Consequently, evaluating the local conductivity on length scales of $\approx 100\ \mathrm{nm}$ is still a well-described quantity. This can for instance be seen in the voltage drop in Figure 4d. Within a MLG/BLG sheet the slope stays constant and does not vary spatially, except at the interfaces and on different sheets at different positions of the sample. For the latter, different local structures of the buffer layer can still lead to different conductivities (averaged on a scale of $\approx 100\ \mathrm{nm}$). This is shown in Fig. 4e in the manuscript.

The absolute resistance values for localized scatterers (Tab. 2 in the manuscript) are in good agreement with Ji et al.[17]. While they did not investigate BLG wrinkles, we find that the values of this defect are larger than SiC-steps, but smaller than the ML/BL-interface. We propose that the transport mechanism here can be described by the transmission through a potential barrier induced by a difference in doping as discussed for the SiC-

steps[18]. Since the width of the wrinkle (~20 nm) is larger than for the SiC-step (<2 nm), the higher resistance for a BLG wrinkle is plausible. Since no electronic transition is involved, the magnitude is much smaller than for ML/BL-interfaces.
Additionally, since the data of Ji et al. is taken at 77 K/300 K the defect resistances seem to stay constant across a large temperature range for ML/BL-interfaces and SiC-steps. Consequently, low-temperature effects such as quantum interference[14,16] cannot play a significant role, which have been observed for some grain boundaries in graphene on SiO_2[19].
In contrast, for a potential barrier model for SiC-steps and BLG wrinkles (Supplementary Note 8) the transmission would not show a strict temperature-dependence. Accordingly, this is supported by the independence of the defect resistance with the magnetic field B (Tab. 2 in the manuscript) in contrast to the sheets. Though the conductivity for the sheets given in Tab. 1 in the manuscript is not intrinsically depending on the magnetic field, the magnetic field leads to an increased time an electron needs to spend in the sheets thus increasing the resistance. This is not or insignificantly the case for the localized defects causing their contribution to vanish with magnetic field as seen in Fig. 2a and Fig. 4d in the manuscript.

Supplementary Note 8

Transmission through a potential barrier with transverse magnetic field: To investigate the magnetotransport through a potential barrier as e.g. the SiC-steps and the bilayer wrinkles in the presence of a magnetic field we here treat the problem by wave function matching and by evaluating the transmission T through the barrier. Previously, it was argued that the detachment of the graphene sheet from the substrate is inducing a drastic change in doping, since SiC(0001)-graphene is heavily n-doped by the underlying buffer layer[17]. However, if it was simply a doping induced process, we would expect a quadratic increase, since the undoped graphene sheet would show the same magnetic field behavior as an n-doped one. However, the change in doping is inducing a potential barrier V which is how we describe the defects here. The geometry of the model is depicted in Supplementary Figure 11.

For the conventional problem of transport through a potential barrier of the form

$$\Phi(x) = \begin{cases} V \text{ if } x \in [-\frac{L}{2}; +\frac{L}{2}] \\ 0 \text{ if } x \notin [-\frac{L}{2}; +\frac{L}{2}] \end{cases} \quad (9)$$

the transmission can be written as

$$T = \frac{4\, k_x k_x' a}{a(k_x + k_x')^2 + \sinh^2(\sqrt{a}L)(a + k_x^2)(a + k_x'^2)} \quad (10)$$

With $a = \frac{2m(V-E)}{\hbar^2} + k_y^2 + k_z^2$. Under the influence of an external magnetic field the Hamiltonian has the form

$$H = \frac{1}{2m}(\mathbf{p} - q\mathbf{A})^2 + q\Phi(x) \quad (11)$$

using

$$\mathbf{A}_{\mathrm{I}} = \left(0, -\frac{BL}{2}, 0\right)^{\mathrm{T}}; \; \mathbf{A}_{\mathrm{II}} = (0, Bx, 0)^{\mathrm{T}}; \; \mathbf{A}_{\mathrm{III}} = \left(0, +\frac{BL}{2}, 0\right)^{\mathrm{T}} \quad (12)$$

Here, the numbers indicate the different regions as indicated in Supplementary Figure 11a. We only include an explicit magnetic field dependence via the change in x inside the barrier (region 2). To assure continuity of the wave functions the k-vectors before and after the barrier have to change

$$\mathbf{k}_{\mathrm{F}} = (k_x, k_y) \longrightarrow \mathbf{k}'_{\mathrm{F}} = \left(\sqrt{k_x^2 + \frac{2eBL}{\hbar}k_y - \left(\frac{2eBL}{\hbar}\right)^2}, k_y - \frac{eBL}{\hbar}\right) \quad (13)$$

This leads to the modified transmission probability

$$T = \frac{4\, k_x \sqrt{k_x^2 + \frac{2eBL}{\hbar}k_y - \left(\frac{2eBL}{\hbar}\right)^2}\; a}{a\left[k_x + \sqrt{k_x^2 + \frac{2eBL}{\hbar}k_y - \left(\frac{2eBL}{\hbar}\right)^2}\right]^2 + \sinh^2(\sqrt{a}L)(a + k_x^2)\left(a + k_x^2 + \frac{2eBL}{\hbar}k_y - \left(\frac{2eBL}{\hbar}\right)^2\right)} \quad (14)$$

In front of the barrier the magnetic field leads to a different angle of incidence as shown in Fig. 3a and 3b in the manuscript. This change is relatively small and the angle can be estimated for 6 T by $\theta = \tan^{-1}(E_y/E_x) \approx \tan^{-1}(0.2) = 11.3\,°$ where the ratio E_y/E_x has been estimated from Fig. 2c and 2d. We included it by a simple linear approximation $\theta = (11.3\,°)\,/\,(6\,\mathrm{T}) \cdot B$ to determine the initial components $k_x = k_{\mathrm{F}} \cdot \cos\theta$ and $k_y = k_{\mathrm{F}} \cdot \sin\theta$. The length of the potential was chosen to be $L = 2\,\mathrm{nm}$ and $L = 20\,\mathrm{nm}$ for a SiC-step and a bilayer wrinkle, respectively. We additionally use $a = \frac{(V-E)}{\hbar\, v_{\mathrm{F}}}$ for graphene with $v_{\mathrm{F}} = 1 \cdot 10^6\,\mathrm{m/s}$.[20] Moreover, we choose the Fermi energies $E_{\mathrm{ML}} = 0.44\,\mathrm{eV}$ and $E_{\mathrm{BL}} = 0.3\,\mathrm{eV}$ (See Supplementary Note 5) as well as $E - V = 80\,\mathrm{meV}$ to adjust the electron concentration in the barrier to an intrinsic, 'undoped' level[7]. Since the transmission can be directly connected to the resistance in the Landauer Büttiker formalism[1] by

$$\frac{1}{R} = G = \frac{2\mathrm{e}^2}{\mathrm{h}} MT \quad (15)$$

We define the magnetoresistance as

$$\mathrm{MR}(B) = \frac{T^{-1}(B)}{T^{-1}(0)} - 1 \quad (16)$$

The results for a SiC-step as well as a BLG wrinkle can be found in Supplementary Figure 11c. As can be seen the MR is small and also negative. For SiC steps we find $\mathrm{MR}(6\,\mathrm{T}) \approx -1\,\%$ and for BLG wrinkles $\mathrm{MR}(6\,\mathrm{T}) \approx -4\,\%$. These rather small changes fit to the experimentally found independence on magnetic field compared to the sheet resistance as described in the manuscript.

Thus, the defect resistance does not change much, if the scattering mechanism is induced by quantum tunneling through a potential barrier. Independent on the absolute value of the transmission, it barely changes due to a deflection inside the barrier and for different incident angles. By using Supplementary Equation (15) we followed for the sake of simplicity the Landauer approach including the contribution of the contacts of a ballistic conductor that contains the defect.[1] This has been previously used to describe the transmission of defects in graphene and 2D systems.[21,22] However, excluding the contacts would lead to $G \propto T/(1-T)$, which still yields a small variation with magnetic field.

Supplementary References

1. Datta, S., *Electronic transport in mesoscopic systems* (Cambridge university press, 1997).
2. Pryor, R. W. *Multiphysics Modeling Using COMSOL?: A First Principles Approach, Infinity Science Serie*s (Jones & Bartlett Learning, 2011).
3. Weiss, H., and Welker, H. Zur transversalen magnetischen Widerstandsänderung von InSb. *Zeitschrift für Physik* **138**, 322-329 (1954).
4. Davies, J. H. *The physics of low-dimensional semiconductors: an introduction.* (Cambridge university press, 1997).
5. Kisslinger, F., Ott, C., Weber, H. B. On the Origin of Non-saturating Linear Magnetoresistivity. Preprint at *arXiv:1609.02418* (2016).
6. Isenberg, I., Russell, B. R., Greene, R. F. Improved method for measuring Hall coefficients. *Rev. Sci. Instrum.* **19**, 685-688 (1948).
7. Katsnelson, M. I., Novoselov, K. S., Geim, A. K. Chiral tunnelling and the Klein paradox in graphene. *Nat. Phys*. **2**, 620-625 (2006).
8. Willke, P., Druga, T., Ulbrich, R. G., Schneider, M. A., Wenderoth, M. Spatial extent of a Landauer residual-resistivity dipole in graphene quantified by scanning tunnelling potentiometry. *Nat. Commun.* **6***,* 6399 (2015).
9. Ohta, T. *et al.*, Interlayer interaction and electronic screening in multilayer graphene investigated with angle-resolved photoemission spectroscopy. *Phys. Rev. Lett.* **98**, 206802 (2007).
10. Druga, T. *Graphen auf Siliziumcarbid: elektronische Eigenschaften und Ladungstransport* (Niedersächsische Staats- und Universitätsbibliothek Göttingen, Göttingen, Germany 2014).
11. Willke, P. *et al.*, Local transport measurements in graphene on SiO2 using Kelvin probe force microscopy, *Carbon* **102**, 470-476 (2016).
12. Ristein, J., Mammadov, S., and Seyller, T. Origin of doping in quasi-free-standing graphene on silicon carbide. *Phys. Rev. lett.* **108**, 246104 (2012).
13. Hibino, H., Mizuno, S., Kageshima, H., Nagase, M., Yamaguchi, H. Stacking domains of epitaxial few-layer graphene on SiC(0001). *Phys. Rev. B* **80**, 085406 (2009).
14. Jobst, J., Waldmann, D., Gornyi, I. V., Mirlin, A. D., Weber, H. B. Electron-Electron Interaction in the Magnetoresistance of Graphene. *Phys. Rev. Lett.* **108**, 106601 (2012).

15. Mahmood, A. *et al.*, Epitaxial graphene morphologies probed by weak (anti)-localization. *J. Appl. Phys.* **113**, 083715 (2013).
16. Willke, P. *et al.*, Doping of graphene by low-energy ion beam implantation: structural, electronic, and transport properties. *Nano Lett*. **15**, 5110-5115 (2015).
17. Ji, S.-H. *et al.*, Atomic-scale transport in epitaxial graphene. *Nature Mat.* **11**, 114–119 (2011).
18. Low, T., Perebeinos, V., Tersoff, J., Avouris, P. Deformation and scattering in graphene over substrate steps. *Phys. Rev. Lett.* **108**, 096601 (2012).
19. Yu, Q. *et al.*, Control and characterization of individual grains and grain boundaries in graphene grown by chemical vapour deposition. *Nat. Mater.* **10**, 443–449 (2011).
20. Neto, A. *et al.*, The electronic properties of graphene. *Rev. mod. Phys.* **81**, 109 (2009).
21. Clark, K. W. *et al.* Spatially resolved mapping of electrical conductivity across individual domain (grain) boundaries in graphene. *ACS nano* **7**, 7956–7966 (2013).
22. Matsuda, I. *et al.*, Electrical resistance of a monatomic step on a crystal surface, *Phys. Rev. Lett.* **93**, 236801 (2004).
